# Combined Effect of Cameo2 and CBP on the Cellular Uptake of Lutein in the Silkworm, *Bombyx mori*

**DOI:** 10.1371/journal.pone.0086594

**Published:** 2014-01-27

**Authors:** Wei Wang, Mao-Hua Huang, Xiao-Long Dong, Chun-Li Chai, Cai-Xia Pan, Hui Tang, Yan-Hong Chen, Fang-Yin Dai, Min-Hui Pan, Cheng Lu

**Affiliations:** 1 State Key Laboratory of Silkworm Genome Biology, Southwest University, Chongqing, China; 2 Key Laboratory for Sericulture Functional Genomics and Biotechnology of Agricultural Ministry, Southwest University, Chongqing, China; 3 College of Animal Science and Technology, Southwest University, Chongqing, China; Wuhan Bioengineering Institute, China

## Abstract

Formation of yellow-red color cocoons in the silkworm, *Bombyx mori*, occurs as the result of the selective delivery of carotenoids from the midgut to the silk gland via the hemolymph. This process of pigment transport is thought to be mediated by specific cellular carotenoids carrier proteins. Previous studies indicated that two proteins, Cameo2 and CBP, are associated with the selective transport of lutein from the midgut into the silk gland in *Bombyx mori*. However, the exact roles of Cameo2 and CBP during the uptake and transport of carotenoids are still unknown. In this study, we investigated the respective contributions of these two proteins to lutein and β-carotene transport in *Bombyx mori* as well as commercial cell-line. We found that tissues, expressed both *Cameo2* and *CBP*, accumulate lutein. Cells, co-expressed Cameo2 and CBP, absorb 2 fold more lutein (*P*<0.01) than any other transfected cells, and the rate of cellular uptake of lutein was concentration-dependent and reached saturation. From immunofluorescence staining, confocal microscopy observation and western blot analysis, Cameo2 was localized at the membrane and CBP was expressed in the cytosol. What’s more, bimolecular fluorescence complementation analysis showed that these two proteins directly interacted at cellular level. Therefore, Cameo2 and CBP are necessarily expressed in midguts and silk glands for lutein uptake in *Bombyx mori*. Cameo2 and CBP, as the membrane protein and the cytosol protein, respectively, have the combined effect to facilitate the cellular uptake of lutein.

## Introduction

Natural colored silk fabric possesses broad market prospects and valuable economic benefits [1]. As one of the most important natural colored silk-spinning insects in the world, *Bombyx mori* divide into white cocoon strains, yellow-red cocoon strains and green cocoon strains [2]. The yellow-red cocoon strains have varies colored cocoons (including yellow, golden yellow, flesh, pink and rust). The main coloring pigments in yellow-red cocoons are carotenoids [3], [4]. Like many other animals, *Bombyx mori* cannot synthesize carotenoids by themselves [5]. Instead, they can ingest carotenoids from the mulberry leaves to produce yellow-red silk [6], [7], [8], [9]. However, the mechanism of absorption and transport of carotenoids from the midgut into the silk gland via hemolymph is still unclear.

Genetic analysis of *Bombyx mori* shows that the formation of yellow cocoon is mainly controlled by yellow blood gene (*Y*), yellow inhibitor gene (*I*) and yellow cocoon gene (*C*). *Y* controls the absorption of carotenoids from the lumen into the epithelium in midgut [10], [11], [12]. *I* inhibits the transport of carotenoids from the midgut epithelium into hemolymph [10], [12]. *C* regulates the delivery of carotenoids from hemolymph into the silk gland [4]. The yellow cocoons appear only in [*Y+^I^C*] mutants [8], [12]. Carotenoid-binding protein (CBP), a product of *Y*, directly binds carotenoids and promotes their transport in *Bombyx mori* [8], [13], [14]. In yellow cocoon strains, CBP is expressed in the midguts and the middle silk glands, and their hemolymph, silk glands and cocoons all appear yellow color [13], [15]. In +*^Y^* allele strains, due to absence of the CBP protein, the midgut epithelium cannot absorb and transport carotenoids into hemolymph or silk glands, resulting in colorless hemolymph, colorless silk glands and white cocoons [10], [13], [14]. *C* locus associated membrane protein homologous to a mammalian HDL receptor 2 (Cameo2), a product of *C*, determines the selective transport of lutein from hemolymph into the middle silk gland [9]. In yellow cocoon strains, both *Cameo2* and *CBP* are expressed in midguts and silk glands [9], [10], [13], implying their potential function of carotenoids transport in specific tissues. *Cameo1* is the only homologous gene of *Cameo2* on chromosome 12, where the *C* locus lies [9]. But it is still unknown whether Cameo1 participates in the cellular uptake of carotenoids in *Bombyx mori*.

Previous studies proposed a hypothesis that Cameo2 might serve as the lutein transporter on the plasma membrane to regulate the transmembrane transport of lutein; and CBP might serve in the cytosol to mediate lutein diffusion in the cytosol [9]. Thus, it is important to investigate the relative contributions of these two proteins to carotenoids transport in *Bombyx mori*. In this study, we explored the mRNA expressions of *Cameo1*, *Cameo2* and *CBP,* associated with carotenoids accumulation in midguts, hemolymph, silk glands and cocoons from *Bombyx mori*. Meanwhile, we examined their contributions to the cellular uptake of carotenoids by using cell-line *ex vivo*. As the result, both *CBP* and *Cameo2* are required in tissues to fulfill lutein accumulation in *Bombyx mori*. And they have combined effects on the uptake and transport of cellular lutein.

## Materials and Methods

### Insect Material

**Figure 1:**
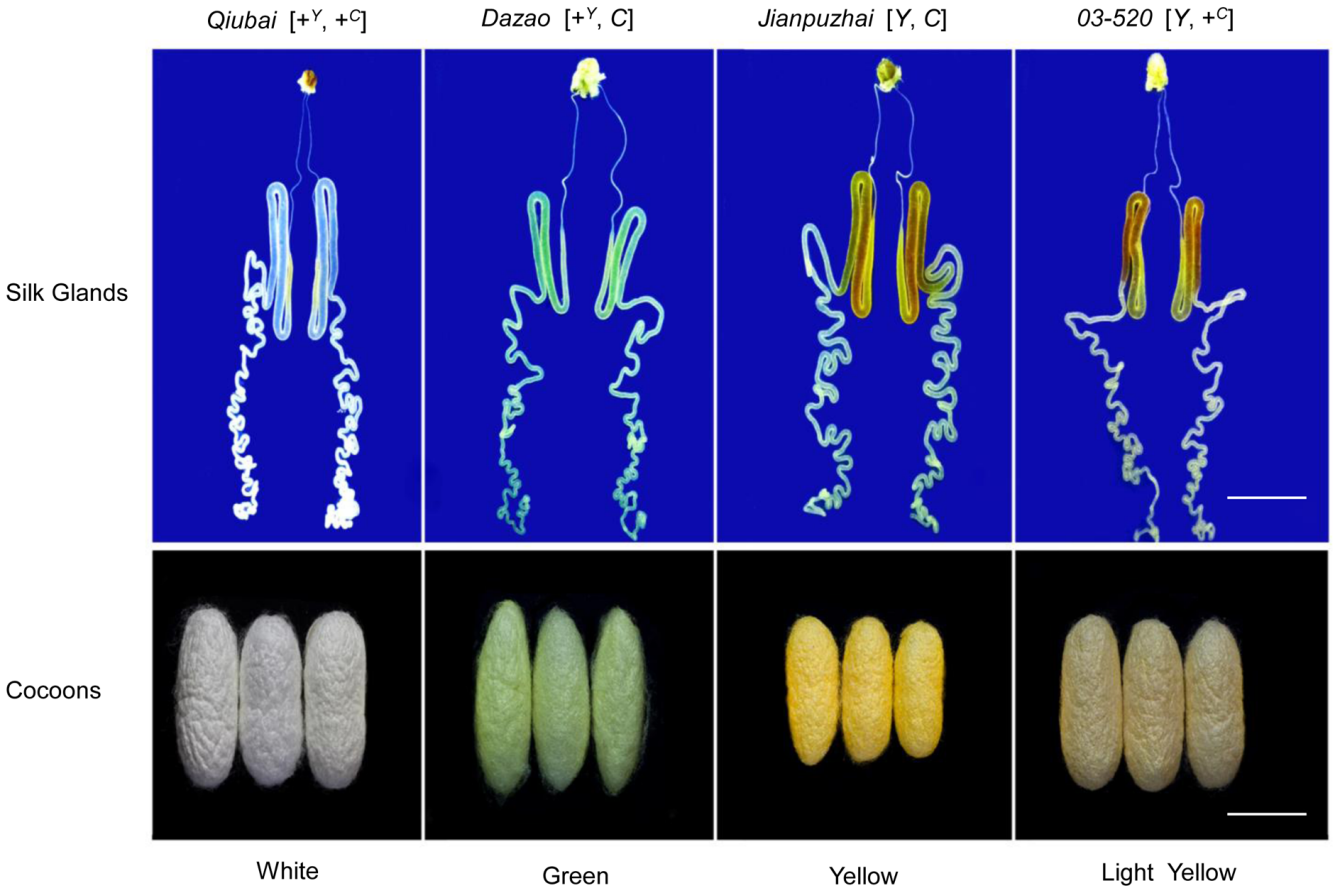
The Genotypes and Colors of Silk Glands and Cocoons in *Bombyx mori*. The silk glands from *Qiubai*, *Dazao*, *Jianpuzhai* and *03-520*, were obtained from last instar larvae stage at 5 days of age. The colors of silk glands are similar as the colors of cocoons from the same strains. The white bars indicate 1 cm.

*Bombyx mori* strains were preserved in the Silkworm Gene Bank at Southwest University, China. The larvae were reared on fresh mulberry leaves until the last instar larvae at 25°C under 12 h light, 12 h dark cycles. Four *Bombyx mori* strains with different colors of cocoons were used: *Qiubai* (colorless silk glands and white cocoons) was used as the genotype of [+*^Y^*, +*^C^*]. *Dazao* (green silk glands and green cocoons) has the genotype [+*^Y^*, *C*] (the type of *Y* mutant is unknown [14]), *Jianpuzhai* (yellow silk glands and deep yellow cocoons) has the genotype [*Y*, *C*] (It is not clear as to how many copies/genome of *Y* [14]) and *03-520* (yellow silk glands and light yellow cocoons) was used as the genotype of [*Y, +^C^*] (Figure 1). Midguts, hemolymph and silk glands were obtained from last instar larvae stage at 3, 4, 5 and 6 days of age and stored at −80°C until use.

### Semiquantitative RT-PCR Analysis

Total RNA was isolated from midguts and silk glands of last instar *Bombyx mori* larvae stage at 3, 4, 5 and 6 days of age by using Trizol reagent (Invitrogen, USA). Reverse transcription was performed by using Oligo (dT) primer (BBI, China) and M-MLV reverse transcriptase (Promega, USA). Then cDNA was used as a template to test *Cameo1* (GenBank Accession No. AB515345.1), *Cameo2* (AB515346.1), *CBP* (AB263201.1) and *actin A3* (A3, NM_001126254). All the primers were designed by Primer Premier 5.0 software (PREMIER Biosoft, USA; Table 1) and purchased from Invitrogen (China). Each tissue was tested in triplicate for total RNA isolation, cDNA synthesis and RT-PCR.

**Table 1:** PCR Primers for RT-PCR and the Construction of pcDNA3.1 B/pEGFP-N1/pBiFC Expression Vectors.

Reaction Type	Gene Name	Primers (5′→3′)	Product Size (bp)	Tm (°C)
RT-PCR	*actin A3*	F: AACACCCCGTCCTGCTCACTG	666	55
		R: GGGCGAGACGTGTGATTTCCT		
RT-PCR/pcDNA3.1 B	*Cameo1*	F: TTCGGGGTACCATGGAGATGGTGTCTTCCGGAG	1485	60
		R:TCTAGTCTAGAGATTGTTGATTCTCTTGTGACGCAC		
	*Cameo2*	F: TTCGGGGTACCATGGGTGGTGGTTTGTTGAG	1482	60
		R: TCTAGTCTAGAGATATAGGATTCAGTTTCATTTCCGC		
	*CBP*	F: TTCCCAAGCTTATGGCCGACTCTACGTCGAAAAG	891	64
		R: TCTAGTCTAGAGAGAATTCGGCTCTGGCCTTCG		
pcDNA3.1 B	*cbp*	F: TTCCCAAGCTTATGGCGAACGCTTGGCG	654	63
		R: TCTAGTCTAGAGAGAATTCGGCTCTGGCCTTCG		
	*EGFP*	F: TTCGGGGTACCATGGTGAGCAAGGGCGAG	717	58
		R: TCTAGTCTAGAGACTTGTACAGCTCGTCCATGCC		
pEGFP-N1	*Cameo2*	F: TTCCGGAATTCCCCGTGGTGGTTTGTTGAGATG	1479	60
		R: AACCGCTCGAGCTATAGGATTCAGTTTCATTTCCGCT		
	*CBP*	F: TTCCCAAGCTTGCCGACTCTACGTCGAAAAGC	888	60
		R: CCTAGTCTAGAGAATTCGGCTCTGGCCTTC		
pBIFC-VC155	*Cameo1*	F: TTGGAAGATCTGCAAGATGGTGTCTTCCGGAGT	1482	58
		R: TTCGGGGTACCTTGTTGATTCTCTTGTGACGCA		
	*Cameo2*	F: TTCCGGAATTCCCCGTGGTGGTTTGTTGAGATG	1479	60
		R: AACCGCTCGAGCTATAGGATTCAGTTTCATTTCCGCT		
pBIFC-VN173	*CBP*	F: TTCCCAAGCTTGCCGACTCTACGTCGAAAAGC	888	60
		R: CCTAGTCTAGAGAATTCGGCTCTGGCCTTC		
	*cbp*	F:TTCCCAAGCTTGCGAACGCTTGGCGC	651	59
		R: CCTAGTCTAGAGAATTCGGCTCTGGCCTTC		

F: forward primer; R: reverse primer.

### Construction of Expression Vectors

**Figure 2:**
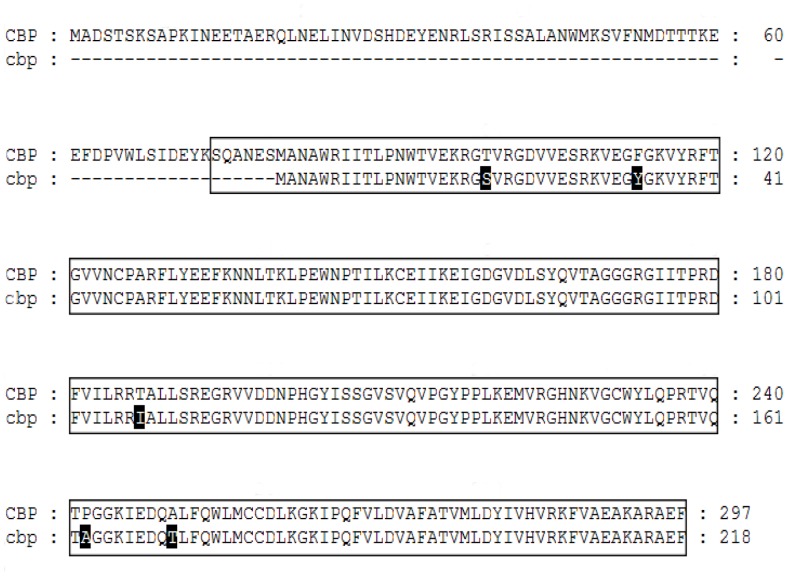
Protein Sequence Comparison Between CBP and cbp. There was an absence of 79 amino acids on the N-terminal of cbp compared to CBP. The boxed area represents the region of steroidogenic acute regulatory protein (StAR)-related lipid transfer (START) domain. The black highlight areas indicate mutation of amino acids residues in cbp compared to CBP.

The pEGFP-N1, pcDNA3.1/V5-His B (pcDNA3.1 B), pBiFC-VC155 and pBiFC-VN173 vectors were used in this study (obtained from Dr. Hong-Juan Cui, State Key Laboratory of Silkworm Genome Biology, Southwest University, China). By sequence alignment of *CBP* in Silkworm Genome Database (http://silkworm.swu.edu.cn/silkdb/), a truncated *CBP* was found and named as *cbp* (Gene ID: BGIBMGA009791-TA). Comparing to CBP protein structure, cbp lacks the 79 amino acids on N-terminal, and has 5 mutations in amino acids residues, resulting in an incomplete steroidogenic acute regulatory protein (StAR)-related lipid transfer (START) domain (Figure 2). In this study, cbp was used as non-functional substitute of CBP. *Cameo1*, *Cameo2*, *CBP*, *cbp* and *EGFP* (Accession No. KC897090) were cloned, cleaved and ligated into different expression vectors by using different primers (Table 1) and restriction endonucleases (Takara, Japan; Table 2).

**Table 2:** Restriction Endonucleases for the Construction of pcDNA3.1 B, pEGFP-N1, pBiFC-VC155 and pBiFC-VC173 Vectors.

		Restriction Endonucleases					
Vector	GeneName	*KpnI*	*XhoI*	*HindIII*	*XbaI*	*EcoRI*	*BglII*
pcDNA3.1 B	*Cameo1*	+			+		
	*Cameo2*	+			+		
	*CBP*			+	+		
	*cbp*			+	+		
	*EGFP*	+			+		
pEGFP-N1	*Cameo2*		+			+	
	*CBP*			+	+		
pBiFC-VC155	*Cameo1*	+					+
	*Cameo2*		+			+	
pBiFC-VN173	*CBP*			+	+		
	*cbp*			+	+		

+: restriction endonucleases used to cleave the PCR products.

### Cell Culture and Transient Transfection

Human embryonic kidney 293 (HEK293) cells (obtained from Dr. Xu Wei, the College of Biological Engineering, Chongqing University, China) were grown in Dulbecco's Modified Eagle Medium (DMEM; Hyclone, USA) with 10% fetal bovine serum (FBS; Gbico, USA) and incubated at 37°C in 95% O_2_/5% CO_2_. One day before transfection, HEK293 cells were seeded at a density of 0.5×10^5^–2×10^5^ cells/cm^2^ on glass cover slips (Fisher Scientific, USA) in 24-well plates (Corning Incorporated, USA) or 6-well plates (Corning Incorporated). Transient transfection was achieved by using the X-tremeGENE HP DNA Transfection Reagent (Roche, USA) according to the manufacturer’s instruction. Each milliliter of medium contained a 2 µg expression vector and 4 µL transfection reagent.

### Analysis of the Cellular Carotenoids Uptake

Carotenoids-rich micellar culture medium was prepared according to the “Tween” method [16], [17]. Briefly, in a sterilized glass tube, carotenoids were dissolved in n-hexane and dried with nitrogen gas. The residue was re-dissolved in Tween 40:acetone (1∶5, v:v). After the solvents were evaporated, the dried residue was solubilized in DMEM containing 10% FBS to obtain a final concentration of 1 to 16 µM carotenoids and 0.1% Tween 40.

Recombinant expression vectors of *Cameo1*, *Cameo2*, *CBP* and *cbp* with His tag were transiently transfected into HEK293 cells with various combinations. At 36 h after transfection, all transfected cells were incubated in medium containing 10 µM carotenoids for 10 h. To determine lutein absorption kinetics, the transfected cells expressing Cameo2+CBP or EGFP were incubated in medium containing 10 µM lutein for 1, 2, 4, 8 and 16 h. Meanwhile, to investigate the relationship between the concentration and the absorption rate of lutein, the transfected cells were incubated in medium containing 1, 2, 4, 8 and 16 µM lutein for 10 h. In this study, the HEK293 cells expressing EGFP were used as control. After incubation, the transfected cells were washed twice with 1×PBS containing 0.1% Tween 40. Then, the cells were harvested and broken using an ultrasonic processor (Beidi-II YJ, China). After measured protein concentration by Bradford protein assay, the isolated proteins were used for western blot analysis. Carotenoids were extracted from the cell lysate and analyzed by high performance liquid chromatography (HPLC).

### Western Blot Analysis

Protein samples from transfected cells were separated by 12.5% SDS-PAGE. The electrophoresed proteins were transferred to the polyvinylidene fluoride membrane, and blocked in 5% non-fat dry milk dissolved in TBST (0.01 M Tris-HCl, 0.15 M NaCl, 0.05% Tween 20, pH 8.0) at 4°C overnight. After washing three times with TBST, the membrane was incubated with the mouse monoclonal anti-His primary antibody (1∶1000, Beyotime, China) and with or without anti-EGFP antibody (1∶1000, Beyotime). Immunodetection was performed using the peroxidase-conjugated anti-mouse secondary antibody (1∶5000, Beyotime). The immunoblot was visualized by using ECL Plus Western Blotting Detection Reagents (GE Healthcare, USA).

### Extraction and HPLC Analysis of Carotenoids from Tissues, Cocoons and Transfected Cells

To clarify the correlation between carotenoids accumulation and the gene expression of *CBP, Cameo1 and Cameo2,* we first measured the carotenoids content in midguts, hemolymph, silk glands and cocoons from four *Bombyx mori* strains by HPLC. Tissues were ground within liquid nitrogen, weighed and placed in a 50 mL centrifuge tube containing the mixture of n-hexane:ethanol:acetone (2∶1:1, v:v:v). The tissue sample was sonicated at 5–10°C for 15 min and centrifuged at 6800×*g* for 10 min. The upper layer extract and the ether extract of the lower layer residual solution were collected into another centrifuge tube. The same sample was re-extracted two times, according to the same protocol as described above. Then, all the extracts were combined and dried by using a lyophilizer (Christ ALPHA 1–4/2–4 LSC, German). The dried residue was dissolved in 2 mL methyl tert-butyl ether (MTBE) containing 0.01% butylated hydroxytoluene (BHT) and 2 mL mixture of KOH: methanol (1∶9, w:v). After over 10 h in darkness, 2 mL MTBE was added to the mixture, then the upper extract was collected and dried. This dried residue was re-dissolved in MTBE containing 0.01% BHT and filtered through 0.45 µm polyvinylidene fluoride filter. Filter samples were used for carotenoids analysis. Each tissue was prepared for three different extract, and each extract sample was measured three times by HPLC.

Each cocoon was cut into small pieces of less than 1 mm width, transferred in a 50 mL centrifuge tube containing 15% NaCO_3_ and sonicated at 60°C for 30 min. Subsequent procedure was followed the same method as tissue preparation. For transfected cells, the cells were washed twice with 1×PBS containing 0.1% Tween 40, and then transferred into a 5 mL centrifuge tube containing 1×PBS. The sample was sonicated at 4°C for 5 min, then added 0.8 mL methanol, 1.2 mL acetone and 1 mL n-hexane. After collected the supernatant, the same sample was re-extracted twice according to the same steps as described above. These extracts were combined, dried and re-dissolved in 50–100 µL MTBE containing 0.01% BHT.

For qualitative and quantitative analysis of carotenoids by HPLC, each combined extract sample (20 µL) was injected into a reverse-phase HPLC system (Agilent1260, USA), consisting of a G1329B auto sample injector, a G1316B quatpump, a G1316A temperature column chamber, a G1315D photodiode array detector and a YMC carotenoid C_30_ column (250 mm×4.6 mm i.d, Japan). The flow rate was 1 mL/min. The gradient elution method consisted of an initial 10 min of 71.2% acetonitrile, 23.8% methanol, 5.0% H_2_O, and 0% MTBE, followed by a linear gradient of 19.5% acetonitrile, 6.5% methanol, 0% H_2_O, and 74.0% MTBE for 31 min. Carotenoids were measured at 450 nm and identified by their retention time and by a spectral analysis (300–700 nm) that compared samples with pure (>95%) standards of all-trans-lutein (Sigma, USA) and all-trans-β-carotene (Sigma, USA). By comparing peak area with standard reference curves, quantification was analyzed with Agilent ChemStation software (version B.04.02, USA). All solvents used for HPLC analysis were HPLC grade (Fisher Scientific).

### Subcellular Localization of Cameo1, Cameo2, CBP and cbp Analysis

To understand the roles and relationships among Cameo1, Cameo2, CBP and cbp for transmembrane transport of carotenoids, prediction of transmembrane helices of these proteins was performed by using TMHMM Server v. 2.0 (http://www.cbs.dtu.dk/services/TMHMM/) [18], [19].

We applied immunofluorescence staining and laser scanning confocal microscopy (LSCM) to determine subcellular locations of those proteins. Briefly, recombinant expression vectors with His or EGFP tag were transfected into HEK293 cells. At 24 h after transfection, cover slips from the 24-well plates were washed with 1×PBS three times. Then, cells were fixed with 4% paraformaldehyde for 15 min, and washed with PBST (1×PBS containing 0.3% Tween 20). Cells were permeabilized with PBST containing 0.1% Triton X-100 for 15 min at room temperature. After washed with PBST three times, cells were blocked with 1×PBS containing 1% BSA and 10% goat serum at 37°C for 1.5 h. Then, cells were incubated with PBST containing anti-His primary antibody (1∶500, Beyotime) at 37°C for 1 h. After washed three times with PBST, cells were incubated with PBST containing Cy3 conjugated rabbit anti-mouse secondary antibody (1∶500, Beyotime) at 37°C for 1 h. After washed three times with PBST, cells were incubated in 4′, 6-diamidino-2-phenylindole (DAPI, Beyotime) for 10 min in darkness. At last, cells were washed six times with PBST and mounted on microscope slides (Citoglas, China). Locations of those proteins in the transfected HEK293 cells were determined by laser scanning confocal microscope (Olympus X81, Japan) at 488 nm and 565 nm.

To further confirm the locations of Cameo1, Cameo2, CBP and cbp proteins in the transfected HEK293 cells, membrane proteins and cytosol proteins were isolated by following the method as described previously [20]. After the bicinchoninic acid (BCA) protein assay for protein quantitation, the locations of Cameo1, Cameo2, CBP and cbp proteins could be determined either on cellular membrane or in cytosol by western blot method as described above.

### Bimolecular Fluorescence Complementation (BiFC) Analysis [21]

In order to test the interaction between Cameo2 and CBP, we inserted either *Cameo1* or *Cameo2* into pBiFC-VC155 vector, and inserted either *CBP* or *cbp* into pBiFC-VN173 vector. Then, these recombinant vectors were transfected into the HEK293 cells. At 24 h after transfection, cells were fixed, permeabilized and staining as described above. Fluorescent images were visualized and digitally captured on a fluorescence microscope system (Olympus BX51). Yellow fluorescence was used to represent the protein-protein interaction between two proteins.

### Statistical Analysis

All data were analyzed by using PASW Statistics 18.0 (SPSS 18.0; IBM, USA) and presented as means ± SEM. Relationships between two variables were examined by one-way ANOVA, with a significance level at *P*≤0.05. The chosen regression was that with the highest squared value of the regression coefficient (*R^2^*).

## Results

### Carotenoids Content and mRNA Expressions of Cameo1, Cameo2 and CBP in Midguts, Hemolymph, Silk Glands and Cocoons

**Figure 3:**
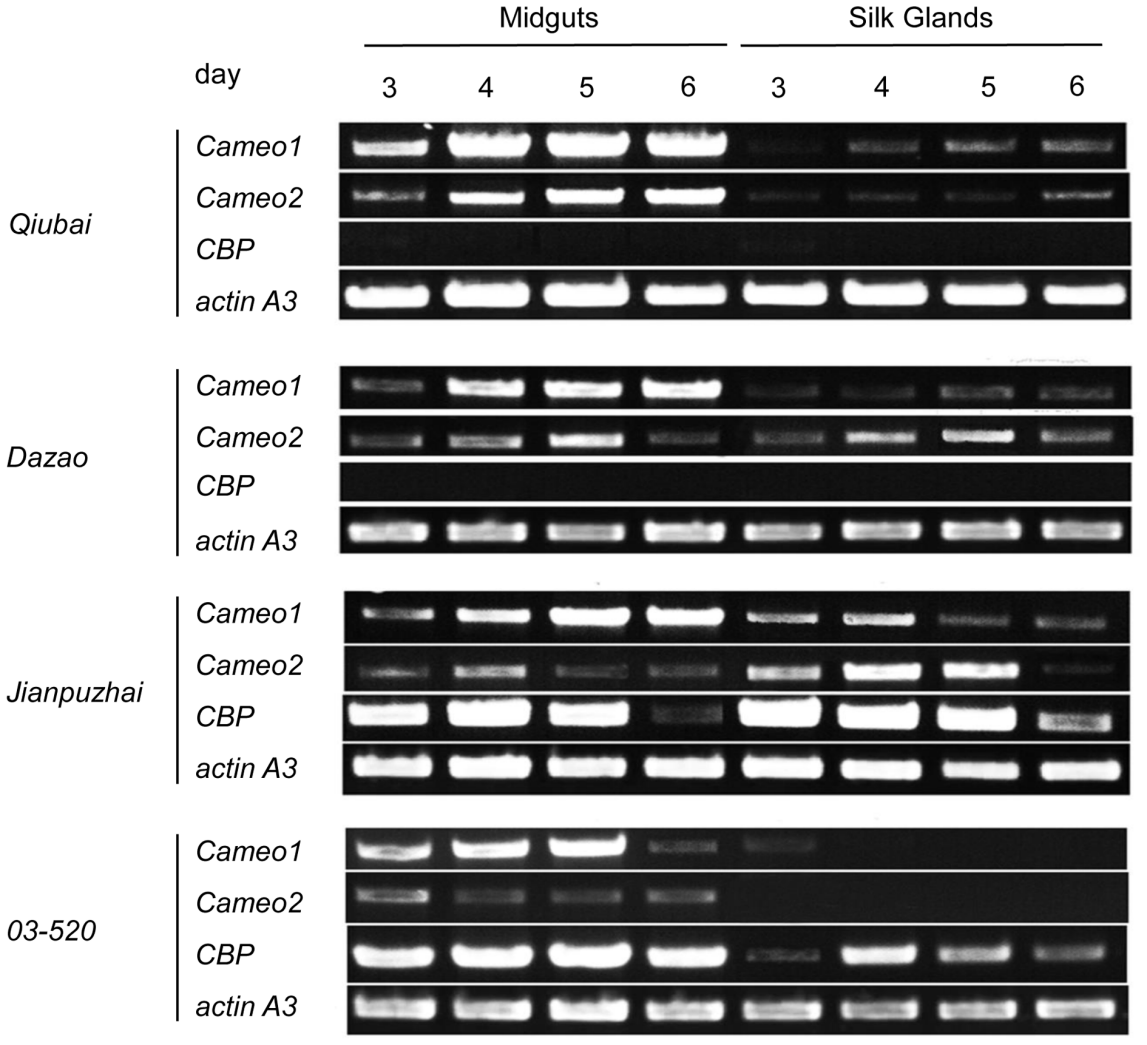
The mRNA Expressions of *Cameo1*, *Cameo2*, *CBP* and *cbp* in Midguts and Silk Glands in *Bombyx mori*. The midguts and silk glands were obtained from last instar larvae stage at 3, 4, 5, and 6 days of age. *CBP* mRNA was expressed in both midguts and silk glands from *Jianpuzhai* and *03-520*; *Cameo2* mRNA was expressed in midguts and silk glands from all strains except the silk glands from *03-520.*

In the current study, gene mRNA expression was determined in midguts and silk glands from four different *Bombyx mori* stains by RT-PCR (Figure 3). The mRNA expressions of *Cameo1* and *Cameo2* were presented in midguts and silk glands from *Qiubai* [+*^Y^*, +*^C^*], *Dazao* [+*^Y^*, *C*] and *Jianpuzhai* [*Y*, *C*]. In *03-520* [*Y*, +*^C^*], both *Cameo1* and *Cameo2* mRNA were expressed in midguts, but only *Cameo1* mRNA existed in silk glands from last instar larvae stage at 3 days of age. *CBP* mRNA was expressed in both midguts and silk glands from *Jianpuzhai* and *03-520*; however, no *CBP* mRNA was detected in *Qiubai* and *Dazao*.

**Figure 4:**
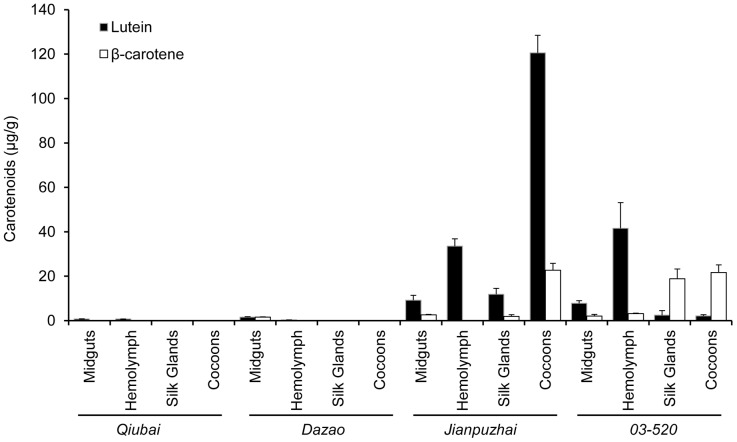
Carotenoids Content in Tissues and Cocoons from *Qiubai, Dazao, Jianpuzhai and 03-520* strains. The midguts, hemolymph and silk glands were obtained from last instar larvae stage at 5 days of age. In these four *Bombyx mori* strains, the types of major carotenoids in silk glands were consistent with cocoons. *Qiubai* and *Dazao* hardly showed carotenoids in their hemolymph, silk glands and cocoons. The major coloring pigment in silk glands and cocoons from *Jianpuzhai* was lutein, but *03-520* was β-carotene.

The levels of two major carotenoids, lutein and β-carotene, were measured in midguts, hemolymph, silk glands and cocoons by HPLC (Figure 4). In *Jianpuzhai*, lutein was the major coloring pigment in midguts, hemolymph, silk glands and cocoons (68.9±3.0%, 77.7±2.2%, 67.2±7.4% and 60.7±2.6% of total carotenoids, respectively). In *03-520*, the ratio of lutein in total carotenoids was significantly higher (*P*<0.05) in midguts and hemolymph (41.9±5.4% and 74.7±7.8%, respectively) compared to silk glands and cocoons (9.9±6.0% and 8.6±2.9%, respectively); On the other hand, the ratio of β-carotene in total carotenoids was higher (*P*<0.01) in silk glands and cocoons (82.0±8.2% and 90.8±3.5%, respectively) than in midguts and hemolymph (11.2±4.6% and 6.1±2.2%, respectively). *Qiubai* and *Dazao*, both of them lack *CBP* mRNA, hardly showed carotenoids in all four tissues. In general, midguts and silk glands, which possess *Cameo1*, *Cameo2* and *CBP* mRNA, have greater ratio of lutein in total carotenoids in *Bombyx mori* (Figure 3 and 4). Tissues, which lack *Cameo2* mRNA, display much less lutein content.

### Carotenoids Concentration in HEK293 Cells Transfected with Cameo1, Cameo2, CBP and cbp

**Figure 5:**
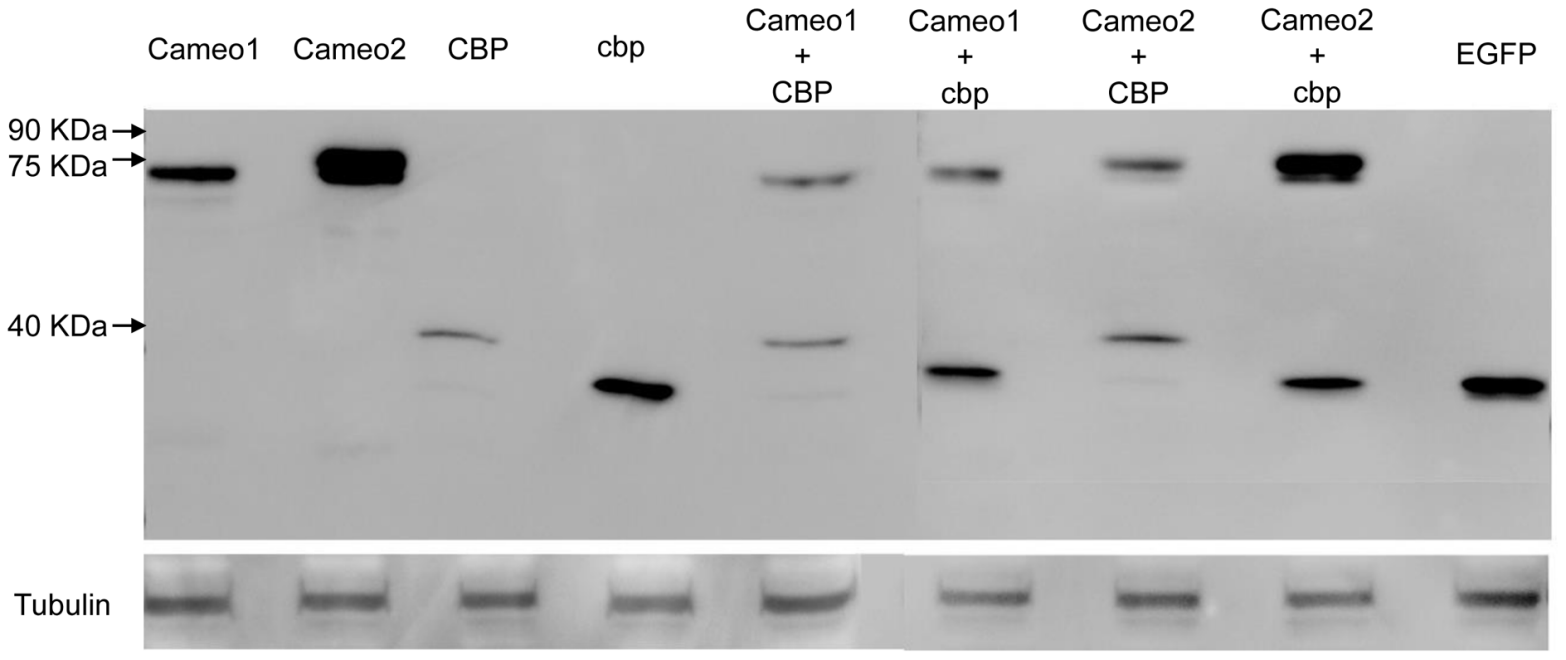
Immunoblots of Cameo1, Cameo2, CBP and cbp in the HEK293 Transfected with Various Combination. Markers of protein molecular weight are indicated on the left.

**Figure 6:**
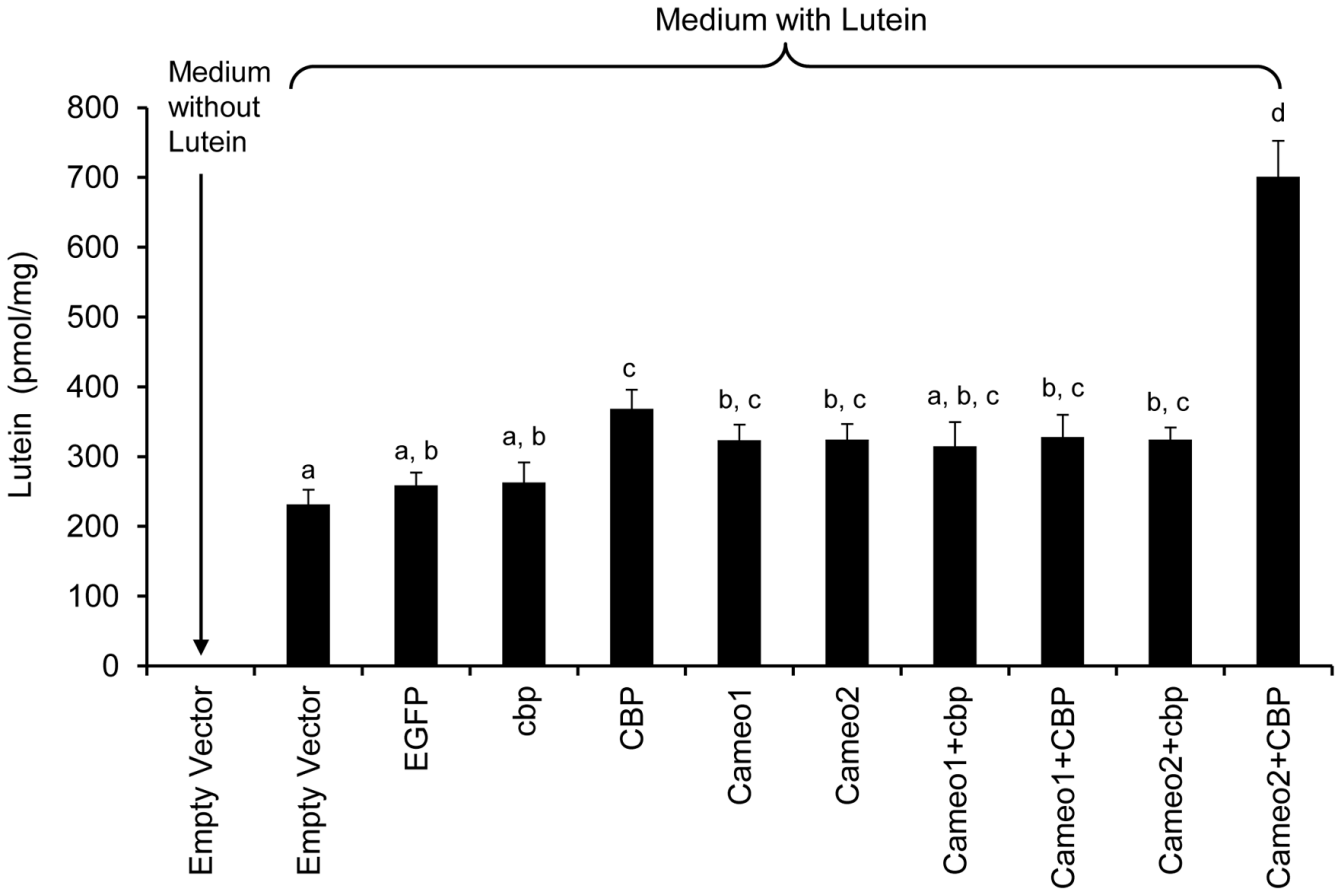
Lutein Concentrations in the HEK293 Cells Transfected with Various Combination of *Cameo1*, *Cameo2*, *CBP* and *cbp*. The cells expressing CBP had slightly higher lutein concentration (*P*<0.05) than the cells transfected with empty vector, *EGFP* and *cbp*. The cells expressing Cameo2 had higher lutein concentration (*P*<0.05) only than the cells transfected with empty vector. The lutein concentration in the cells expressing Cameo2+CBP was 2 fold higher (*P*<0.01) than other transfected cells. Different letters represent significant difference.

In the transfected HEK293 cells, protein expressions of Cameo1, Cameo2, CBP, cbp and EGFP were confirmed by western blot to ensure the accuracy of transfection (Figure 5). Analyzed by HPLC, lutein concentration in the cells expressing EGFP (control) was not different from the cells transfected with empty vector or *cbp* (Figure 6). The cells expressing CBP had slightly higher lutein concentration (*P*<0.05) than the cells transfected with empty vector, *EGFP* and *cbp*. The cells expressing Cameo2 and Cameo2+cbp had higher lutein concentration (*P*<0.05) only than the cells transfected with empty vector. However, lutein concentration in the cells expressing Cameo2+CBP was 2.7 fold higher (*P*<0.01) than control and 2 fold higher (*P*<0.01) than other transfected cells. Lutein was not detected in the cells transfected empty vector incubated with non-lutein medium. Conversely, β-carotene concentration in HEK293 cells transfected empty vector was not statistically different from all transfected groups incubated with β-carotene medium (data not shown). There was no detection of β-carotene in the cells transfected empty vector incubated with non-β-carotene culture medium, as well.

**Figure 7:**
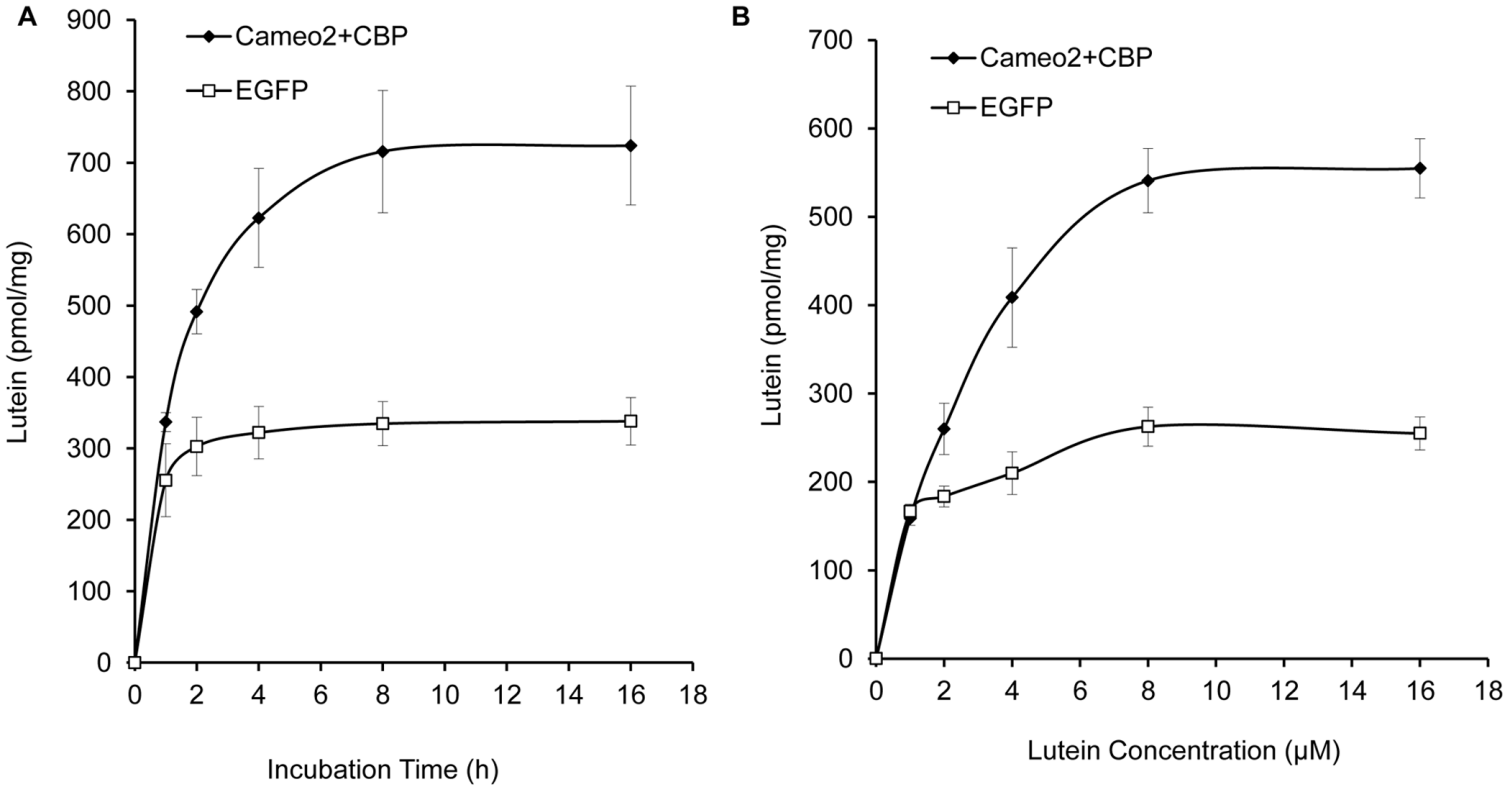
The Absorption Rate of Lutein Associated with Time of Incubation and Concentration Gradient of Lutein in Transfected HEK293 Cells. (A) Time course of cellular uptake of lutein into the transfected HEK293 cells. The lutein absorption rate in the cells expressing Cameo2+CBP increased with the prolonged incubation time and achieved plateau after 8 h incubation. (B) Effect of lutein concentration on the cellular uptake of lutein. The absorption rate of lutein was positively related to the lutein concentration in cells expressing Cameo2+CBP, and reached plateau at higher lutein concentration (8–16 µM).

In order to analyze the characteristics of lutein absorption, we incubated the HEK293 cells expressing Cameo2+CBP or EGFP in lutein-rich medium for different periods of time. The absorption rate of lutein increased rapidly during the first 4 h incubation, and then slowed down over time and achieved plateau after 8 h incubation (Figure 7A). In the cells expressing Cameo2+CBP, the time depended trend of lutein absorption rate could be best described by S curve: Y = e ^(6.65–0.84/x)^ (N = 5, *R^2^* = 0.995, *P*<0.01). Moreover, the absorption rate of lutein was positively related to the lutein concentration in medium, and plateaued at higher lutein concentration (8–16 µM). The concentration depended trend of lutein absorption rate was best described as Y = e^(6.386–1.381/x)^ (N = 5, *R^2^* = 0.974, *P*<0.01; Figure 7B).

### Subcellular Localization and Protein-protein Interaction of Cameo2 and CBP during Carotenoids Transport

**Figure 8:**
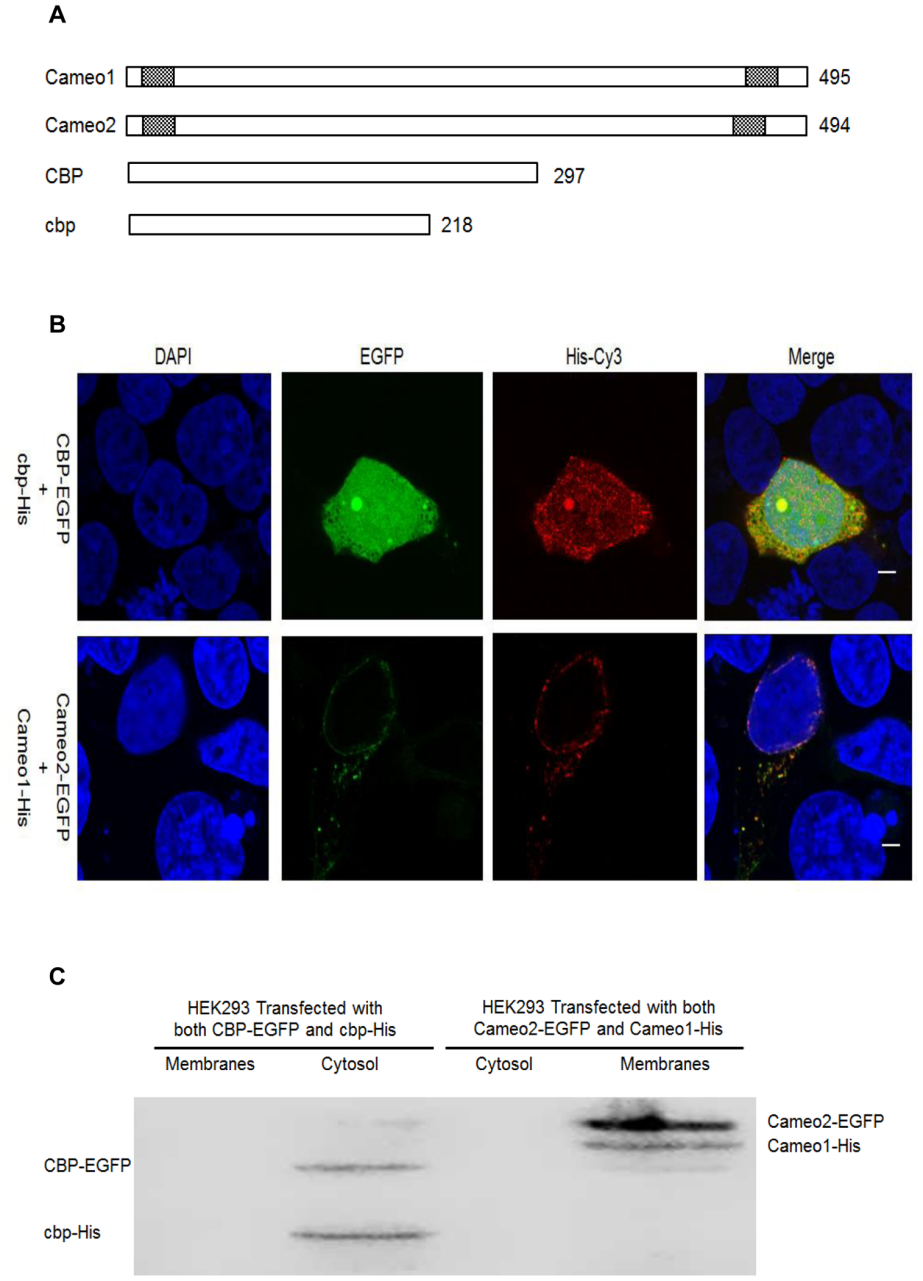
Subcellular Localization of Cameo1, Cameo2, CBP and cbp in Transfected HEK293 Cells. (A) Protein primary structures of the Cameo1 Cameo2, CBP and cbp. The grid boxes represent predicted transmembrane domains. Both Cameo1 and Cameo2 contain two transmembrane regions on each near C- and N-terminal and a putative large extracellular domain. The value represents the number of amino acid residues in protein. (B) The fluorescence of HEK293 cells expressing CBP-EGFP+cbp-His and Cameo1-His+Cameo2-EGFP. The labeling of recombinant expressed proteins with His tag was detected with an antibody raised against the His tag epitope of the proteins and Cy3 conjugated secondary antibody (red). Fusion proteins with EGFP tag glows green. Cell nuclei were stained with DAPI (blue). The white bars indicate 5 µm. The fluorescence of Cameo1-His and Cameo2-EGFP was detected only on the plasma and nuclear membranes in transfected HEK293. In contrast, the fluorescence of CBP-EGFP and cbp-His was mainly presented in the cytosol. (C) Immunoblots of CBP-EGFP, cbp-His, Cameo2-EGFP and Cameo1-His in membrane fractions and cytosol fractions from transfected HEK293 Cells. Cameo1 and Cameo2 were expressed only in the membrane-rich fractions, while CBP and cbp were expressed only in cytosol fractions.

**Figure 9:**
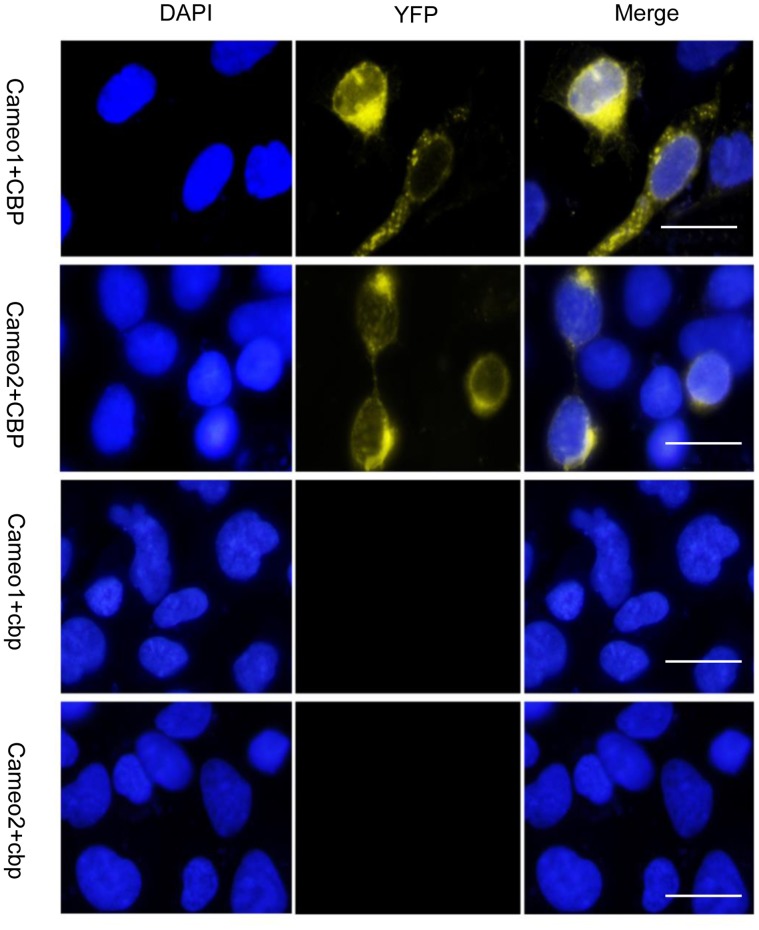
Protein-protein Interactions of Cameo1+CBP and Cameo2+CBP from Bimolecular Fluorescence Complementation Analysis. Cell nuclei were stained by DAPI (blue). Yellow fluorescence indicated that two separated non-fluorescent fragments can interact with each other to form complete yellow fluorescent proteins. The yellow fluorescence was detected in the HEK293 cells expressing Cameo1+CBP or Cameo2+CBP. YFP represents yellow fluorescent protein. The white bars indicate 25 µm.

Predicted protein structures revealed that both Cameo1 and Cameo2 had two transmembrane regions on each near end of C- and N-terminal (Figure 8A). But the protein structures of CBP and cbp did not show any transmembrane domains. From immunofluorescence staining and LSCM, the fluorescence of Cameo1-His and Cameo2-EGFP was detected only on the plasma and nuclear membranes in transfected HEK293 (Figure 8B). In contrast, the fluorescence of CBP-EGFP and cbp-His was mainly presented in the cytosol (Figure 8B). From western blot analysis, immunoblots of Cameo1 and Cameo2 were exhibited only in the membrane-rich fractions, while CBP and cbp were expressed only in cytosol fractions (Figure 8C). BiFC analysis showed that yellow fluorescence was detected in the HEK293 cells expressing Cameo1+CBP or Cameo2+CBP, but not Cameo1+cbp or Cameo2+cbp (Figure 9).

## Discussion

In order to form colored cocoons in *Bombyx mori*, carotenoids from the mulberry leaves must pass though the midgut and entered into the silk gland. This entire process is systematically orchestrated by many factors [3], [12]. Recent studies indicated that Cameo2 and CBP are involved in the transport of carotenoids within larvae of *Bombyx mori* with yellow cocoons [9], [14], [15]. In the current study, the *Jianpuzai* with both *Cameo2* and *CBP* expressed in midguts and silk glands could generate lutein-related yellow cocoons. Without either *Cameo2* or *CBP* expression, lutein cannot be accumulated in silk glands, resulting in other colored cocoons (Figure 1, 3 and 4). After transfected *Cameo1*, *Cameo2*, *CBP* and *cbp* into HEK293 cells with various combinations, lutein concentration in the cells expressing Cameo2+CBP was 2 fold higher (*P*<0.01) than other transfected cells (Figure 6). After incubated in lutein-rich medium, the absorption rate of lutein in transfected HEK293 cells was correlated with time and lutein-concentration until reached saturation (Figure 7A and B). Protein structure prediction, immunofluorescence staining, LSCM and western blot analysis indicated that Cameo2 was the membrane protein, and CBP was only existed in cytosol (Figure 8A, B and C). BiFC analysis showed that Cameo2 had directly protein-protein interaction with CBP at the cellular level (Figure 9). Therefore, these data indicated that Cameo2 and CBP are important regulatory proteins of lutein accumulation during the formation of yellow cocoons in *Bombyx mori*. Cameo2 and CBP, as the membrane protein and the cytosol protein, respectively, have the combined effect to facilitate cellular lutein transport.

From the four strains of *Bombyx mori*, *Jianpuzhai*, which express both *Cameo2* and *CBP*, have lutein-related yellow silk glands and yellow cocoons (Figure 1, 3 and 4). In *03-520*, although *CBP* was expressed in midguts and silk glands, in absence of *Cameo2* in silk glands, the colors of silk glands and cocoons are only associated with β-carotene (Figure 3 and 4). Other strains without *CBP* expression barely have any carotenoids in their hemolymph, silk glands and cocoons (Figure 3 and 4), and the result is similar to previous studies [14], [15]. Thus, in order to form lutein-related yellow cocoons, it requires both *Cameo2* and *CBP* are expressed in midguts and silk glands in *Bombyx mori*.

In order to understand whether Cameo1/2 and CBP directly facilitate lutein accumulation to form yellow cocoons, at first, we measured lutein and β-carotene concentration in the transfected HEK293 cells. As the result, the cells expressing Cameo2+CBP can absorb significantly higher lutein (*P*<0.01) compared to control (Figure 6). However, the lutein concentration of the cells only expressing Cameo1 or Cameo2 was no different from control (Figure 6). What’s more, the cells expressing CBP can absorb 1.42 fold more lutein (*P*<0.05) than control; but the lutein concentration of the cells expressing Cameo1+CBP was not different from control (Figure 6). Therefore, both Cameo2 and CBP are the most important transporters for the cellular absorption and transport of lutein. The accumulation of cellular lutein requires the expressions of Cameo2 and CBP at the same time.

At the cellular level, the rate of lutein absorption in the cells expressing Cameo2+CBP was correlated to the incubation time and the lutein concentration, and reached saturation (Figure 7A and B). This absorption characteristic suggests that the cellular lutein accumulation is regulated by the transmembrane proteins [22]. However, in the transfected HEK293 cells incubated with the β-carotene rich medium, the β-carotene concentration was not different from all the groups (data not show). Thus, the cellular uptake and transport of β-carotene might be controlled by other factors. Sakudoh et al. have identified a Cameo2 homolog, SCRB15, as the β-carotene transporter [23].

In order to investigate the roles of Cameo2 and CBP during transmembrane transport of lutein, we predicted that only Cameo1/2 protein, not CBP/cbp, has two transmembrane regions on each near end of C- and N-terminal (Figure 8A). Base on immunofluorescence staining and LSCM, Cameo1/2 was localized at plasma membranes and nuclear membranes, but CBP/cbp spread in the whole cells (Figure 8B). Meanwhile, immunoblotting analysis confirmed that Cameo1/2 and CBP/cbp proteins existed in isolated membrane fractions and cytosol fractions, respectively (Figure 8C). Thus, Cameo2 and CBP regulate lutein absorption at two separate locations in HEK293 cells. Previous studies showed that Cameo2 is homologous with SR-BI protein (mammalian) and NinaD protein (*Drosophila*) [23]. Both SR-BI and NinaD are membrane proteins [17], [24] and directly mediate the transmembrane transport of carotenoids in Caco-2 TC-7 cells and *Drosophila* S2 cell-line, respectively [25], [26]. Thus, Cameo2 plays the role at the plasma membrane to identify and facilitate lutein into cells.

Besides, CBP contains a unique structural feature of START domain [8], [27] that aids in lipid recognition or transfer [28], [29]. CBP also can be isolated and purified from the cytoplasm of the silk glands of N4 strain and binds lutein with a 1∶1 molar ratio [8]. Moreover, a recent study found that STARD3, a homology of CBP, has specific binding with lutein in the macula of the human retina [30]. Those proteins with the START domain are located primarily in the cytosol, the nucleus and the Golgi rather than in the plasma membrane [28]. Therefore, CBP might act as the cytosolic transporter to bind and transport lutein from plasma membrane into the cytosol.

From BiFC assay, yellow fluorescence from the cells co-expressing Cameo1/2 and CBP indicated both Cameo1 and Cameo2 have the protein-protein interaction with CBP, but not cbp (Figure 9). As the homologous protein of Cameo2, Cameo1 does directly interact with CBP, but it still lacks the regulatory function of lutein transport in cells (Figure 6 and 9). Meanwhile, cbp lacks the ability to interact with Cameo1/2, indicating the absent part of cbp or the mutation of amino acids residues in the START domain (Figure 2) determines essential cellular protein-protein interaction with Cameo2.

In conclusion, the formation of lutein-related yellow cocoons requires the expression of both Cameo2 and CBP in midgut and silk gland in *Bombyx mori*. Cameo2 and CBP are located at the membrane franctions and the cytosol, respectively, and interact with each other to mediate the transmembrane transport of lutein. These findings provide evidence to show that Cameo2, as a membrane protein, is responsible for identifying lutein; CBP, as a cytosolic protein, captures lutein from the plasma membrane and diffuses it in the cytosol.
